# Effects of exercise on life satisfaction of people diagnosed with cancer: a systematic review and meta-analysis

**DOI:** 10.1007/s00520-024-08486-3

**Published:** 2024-04-19

**Authors:** Javier Fernández-Sánchez, Daniel Trujillo-Colmena, Adrián Rodríguez-Castaño, Ana Myriam Lavín-Pérez, Juan Del Coso, Arturo Casado, Daniel Collado-Mateo

**Affiliations:** 1 Sport Sciences Research Centre, Rey Juan Carlos University, Fuenlabrada, Madrid, Spain; 2 GO fitLAB, Ingesport, Madrid, Spain

**Keywords:** Satisfaction with life, Health-related quality of life, Exercise, Oncology, Depression, Survival

## Abstract

**Purpose:**

A cancer diagnosis is commonly associated with a decline in patient’s life satisfaction and more pessimistic expectations about the future. The identification of strategies to improve life satisfaction in patients with cancer is of great interest to health practitioners since it may be associated with a better prognosis of cancer and higher survival rates. Previous meta-analyses and reviews concluded that exercise could significantly improve health-related quality of life in this population, but the effects of exercise on life satisfaction are still not well-known. This review aims to analyse the effects of exercise programs on life satisfaction in people with cancer and individuals who have overcome cancer.

**Methods:**

The present systematic review and meta-analysis followed the Preferred Reporting Items for Systematic Reviews and Meta-analyses (PRISMA) guidelines. A thorough search of databases including Web of Science and PubMed/MEDLINE was carried out. Six studies (535 participants) in which the effect of an exercise program was compared to a non-exercise program control condition in patients with cancer were considered eligible. A subsequent meta-analysis was performed using the random effects model to calculate the standardized mean differences (SMD) and 95% confidence intervals (CI).

**Results:**

Exercise intervention improved satisfaction with life compared with a control condition (SMD = 1.28; *p* = 0.02 with a 95% CI of 0.22 to 2.34).

**Conclusion:**

Exercise could be considered an effective tool to improve life satisfaction in patients with cancer. Hence, professionals might consider the possibility of integrating physical exercise into strategies aimed at enhancing the low life satisfaction often experienced by patients.

**PROSPERO:**

CRD42023438146

## Introduction

Cancer is the second worldwide cause of death, and in 2024, 2,001,140 new cancer cases and 611,720 cancer deaths are estimated to occur in the USA [1]. Despite the high morbidity and mortality rates of several types of cancer, the number of survivors is increasing due to advances in early cancer detection protocols and treatments [2]. In this regard, there is an increasing focus on strategies to enhance not only survival rates but also health-related quality of life (HRQoL) and life satisfaction variables in patients with cancer throughout their lifespan. This is especially important due to the diagnosis is often perceived as an emotional trauma that declines patients’ life satisfaction and induces pessimistic expectations about the future [3]. The decline of overall life satisfaction in persons diagnosed with cancer is aggravated by the associated symptoms of the different types of cancer and the side effects of the most-common treatments [4], including nausea and vomiting [5], diarrhoea [6], sleep disorders [7] and hair loss [8] in the short term and cardiovascular disease [9], sarcopenia [10], cachexia [11] and loss of muscle function and physical function [12] in the long term. Additionally, cancer itself and the treatments employed can also cause other drawbacks as increased fear of death [13] or demoralization [14]. Furthermore, cancer diagnosis is associated with the occurrence of distress [15], less self-esteem [16], depression [17], anxiety [17], pain [18] and cancer-related fatigue [19]. These side effects are negatively correlated with patients’ HRQoL [12], suggesting that cancer is an illness that may influence overall life satisfaction in the years and decades following the diagnosis [20]. Thus, life satisfaction is an important measure of HRQoL and is prospectively related to physical and mental health variables.

Life satisfaction can be defined as an overall evaluation of a person’s HRQoL according to his or her chosen criteria, being a cognitive and critical process [21] in which subjects compare their real life with their ideal life [22]. In patients diagnosed with cancer, life satisfaction may be severally compromised at the beginning of the illness as the well-known negative consequences of cancer ward off patients from an ideal life. Interestingly, some domains of life satisfaction such as social relationships may improve following cancer diagnosis consistent with the concept of the disability paradox, wherein patients demonstrate a capacity for a hedonic adaptation, enabling them to mitigate some of the psychological impact of the diagnosis [23]. Enhancing life satisfaction in healthy and diseased individuals is a crucial variable due to low levels of life satisfaction have been associated with an increased risk of death [24] and a higher risk of suicide [25]. In this sense, the suicide rate of persons with cancer is 1.50 to 1.70 times higher than that in the general population [26]. Overall, scientific evidence suggests that better life satisfaction ratings may positively impact both longevity and HRQoL, particularly in populations that have experienced a recent trauma [3, 24, 27]. For all these reasons, it seems necessary to search strategies for improving life satisfaction among people with cancer as it may have a direct impact on improving prognosis in the short and long term [28].

The link between life satisfaction and HRQoL has been previously studied [27]. However, although there is evidence about the potential effects of regular physical exercise on HRQoL [29–32], its effects on life satisfaction are less clear. In this sense, there are systematic reviews and meta-analyses showing that exercise can improve HRQoL of individuals diagnosed with cancer [29–32]. Regarding the modality of physical exercise, it has been found that both aerobic exercise and resistance exercise seem to be effective in improving HRQoL in individuals diagnosed with cancer [29, 32]. Additionally, there is evidence suggesting that high-intensity exercise may also be effective in enhancing overall HRQoL in individuals diagnosed with cancer [30, 32]. The potential effect of exercise to enhance HRQoL has been investigated across various cohorts of cancer, such as patients with prostate [29], breast [31] or lung cancer [32], among others, which suggests effectiveness of exercise to improve quality of life independently of the type of cancer. Consequently, contemporary clinical perspectives for the treatment of cancer consider exercise programs as an adjunctive therapeutic approach to enhance HRQoL in patients with cancer. However, the life satisfaction variable includes other psychological aspects beyond the perception of health and, to our knowledge, there are no systematic reviews or meta-analyses aimed to evaluate the effects of exercise on the life satisfaction of individuals diagnosed with cancer.

In other populations, such as people of advanced age, empirical evidence suggests that physical exercise could have a significant effect on improving life satisfaction [33]. In people with cancer, higher levels of physical activity (defined as any bodily movement produced by skeletal muscles that results in energy expenditure [34]) are associated with higher levels of life satisfaction [35]. Nevertheless, the link between physical exercise, understood as a subset of physical activity that is planned and structured to obtain an improvement of physical fitness [34], the evidence is unclear regarding its effect on improving life satisfaction in people with cancer. Previous meta-analyses and systematic reviews showed that exercise could reduce depression in patients with breast cancer [36] and anxiety symptoms in patients with breast, prostate, gynaecologic, haematologic and other types of cancer [37]. Other potential benefits of physical exercise in these patients are the reduction of cancer-related fatigue [38] and pain [39], as well as the enhancement of self-esteem [40]. So, although some variables associated with life satisfaction have been shown to be improved with exercise in patients with cancer, the overall effect of regular exercise on life satisfaction has not been properly concluded.

All the mentioned evidence suggests a potential effectiveness of regular exercise on the life satisfaction of persons diagnosed with cancer, but, to the authors’ knowledge, there is a lack of meta-analyses and systematic reviews analysing this. For this reason, the aim of the present systematic review and meta-analysis was to analyse the effects of physical exercise programs on life satisfaction in persons with cancer and individuals who have overcome cancer. Based on the previously documented effectiveness of regular exercise, it was hypothesized that a program of regular exercise would improve life satisfaction in persons with cancer and individuals who have overcome cancer.

## Methods

The present investigation adopted both qualitative (systematic review) and quantitative (meta-analysis) approaches. This systematic review and meta-analysis followed the PRISMA (Preferred Reporting Items for Systematic Reviews and Meta-analyses) guidelines [41]. It was prospectively registered in the International Prospective Register of Systematic Reviews (PROSPERO) with the identification number CRD42023438146.

### Data sources and searches

The search was carried out in the databases Pubmed and Web of Sciences (KCI-Korean Journal Database, MEDLINE, Russian Science Citation Index and SciELO Citation Index). Articles were systematically identified using the search strategy shown in Table 1. The search started on March 1, 2023, and ended on March 17, 2023. Additionally, due to the possibility of new studies during the review process of the manuscript, the search was updated 1 year later, in March 2024. It was conducted without any publication year restriction. Secondary searches consisted of screening the reference lists of the included studies as well as the examination of the papers that have cited the included studies through the Scopus database. Following the removal of duplicates, a two-stage search approach was used. Titles and abstracts were initially reviewed to eliminate irrelevant articles based on eligibility criteria. The full text of the articles included in the first stage was read in the second stage to determine whether the article matched the inclusion criteria (Table 1). The literature search and the articles’ selection process were performed by three independent authors (JFS, DTC, ARC), while disagreements between these authors were settled through discussion with the rest of the investigators (DCM, ALP, JDC and ACA).

**Table 1:** Search strategy and eligibility criteria

Search strategy	Inclusion criteria	Exclusion criteria
("exercise" or "training" or "physical activity")	Participants were patients or survivors of any type of cancer	Participants were under 18 years
(“cancer”)	Physical exercise intervention was performed	Interventions not involving a controlled exercise condition
("satisfaction with life" or "life satisfaction")	The study incorporates a non-exercise intervention control group	The control group has no similar characteristics to the exercise group
	Life satisfaction was reported	Life satisfaction is not reported in quantitative data
	Randomised control trials and non-randomised control trials	Systematic reviews, meta-analyses, case-control studies, study protocols, symposia or congresses

### Risk of bias assessment

The Physiotherapy Evidence Database Scale (PEDro) scale, which is specific to physiotherapy and has been adopted in sport sciences [42], was used. This scale has been previously validated, and it is a reliable tool to assess eligibility, randomisation, blinding and whether the groups are similar at baseline or not [43]. This 11-item scale assesses the internal and external validity of each study. Each included paper is given a score from 1 to 10, depending on whether it meets the items of the PEDro scale. The first item of the scale, which measures the applicability of the trial, is not used for the scoring of the PEDro scale.

In addition, the Evidence Project risk of bias tool was also used to assess the risk of bias in each study. This is a reliable tool for intervention studies with different designs [44]. This tool is composed of eight items, and the overall score ranges from 1 to 8, obtained by adding the number of items that each study fulfils. The items of the Evidence Project risk of bias tool cover the design of the study by assessing whether there is a follow-up- and a control group, and whether pre-values are presented. In addition, it assesses if there is a random selection of the sample and if participants were randomly assigned to each group. Finally, it also assesses whether participants’ socio-demographic characteristics and values were similar between groups at baseline. The first three items assess the study design. The other five items assess elements defining the rigour of the study.

### Data extraction

Following the PRISMA methodology, participants, intervention, comparisons, outcomes and study design (PICOS) were obtained [41] (Table 2). The principal outcome of the current systematic review and meta-analysis was life satisfaction. Thus, the available information reported by the studies regarding this variable was extracted, irrespective of the type of questionnaire employed. In this sense, the different questionnaires employed to measure life satisfaction were collected as well as their scoring requirement process to obtain a better comprehension of the results. This was measured through the “Satisfaction with life scale” [22], the “life satisfaction inventory” [45] and the “D1 subscale of the Campbell questionnaire”. In addition, different data were extracted to characterise the sample and the interventions.

**Table 2:** Data extraction

PRISMA methodology	Data extraction
Participants	Sample, age, gender, cancer type, cancer stage, type of treatments, time when the exercise intervention was applied (before, during or after treatment), level of physical activity, BMI
Intervention	Intervention length, sessions duration, frequency, intensity, exercise modality, type of exercise, adherence (rate of people completing the intervention and rate of attendance to sessions) and main characteristics of the control and the exercise interventions
Comparisons	Exercise and control group
Outcomes	Life satisfaction score
Study design	Control trials (RCTs) and non-RCTs

*BMI* body mass index

### Statistical analysis

Means and SD of pre-to-post-intervention change in life satisfaction scores or, when appropriate, post-intervention life satisfaction scores were extracted from the articles in both control and experimental groups [46]. If articles reported data indicating the change between pre-intervention and post-intervention, it was used. However, in instances where the selected studies omitted data detailing such change, but their design was randomised, the measure of life satisfaction post-intervention was utilized [46, 47]. In non-randomised studies wherein data indicating change were not reported, both pre- and post-intervention life satisfaction values were used for the assessment of such change. In these cases, the SD was calculated using *t* values [46].

Statistical analysis was performed with the Review Manager Software (version 5.3, London, UK). Inverse variance and random effects were used due to the heterogeneity of the articles’ interventions and patients’ characteristics [48]. In addition, standardized mean difference (SMD) was selected because of the use of different life satisfaction scales in the selected studies [46]. Results were reported with a 95% confidence interval (CI). The *p* value fixed to consider the result statistically significant was < 0.05. SMD effect size was interpreted, according to the Cochrane Handbook of Systematic Reviews [49], as small with results < 0.40, moderate 0.40 to 0.70 and large > 0.75 [49].

## Results

### Study selection

**Fig. 1:**
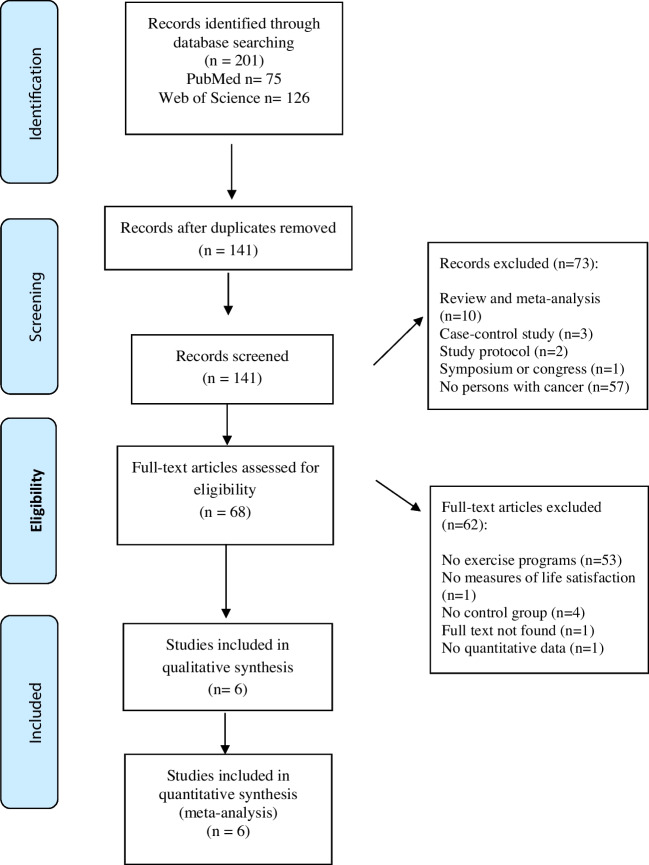
Figure flow chart

Six articles were included in the systematic review and meta-analysis. The flow chart is shown in Fig. 1 and describes the process of inclusion or exclusion of studies.

### Risk of bias

Table 3 shows the PEDro scale. The mean score of the articles was 6.17 with an SD of 1.72, the maximum score being 10. Two studies only received a score of 4, whereas one study achieved a score of 8. Five of the six articles comply with the items of specification of the eligibility criteria and the randomisation of the groups. However, none of them complied with the blinding item because participants knew whether they were either exercising or not.

**Table 3:** Assessment of PEDro scale items of the studies included in the systematic review

Estudio/item	1	2	3	4	5	6	7	8	9	10	11	Total
Campbell et al., 2005	Yes	Yes	Yes	Yes	No	No	No	Yes	Yes	Yes	Yes	7
Courneya et al., 2003	Yes	Yes	Yes	No	No	No	Yes	Yes	Yes	Yes	Yes	7
Kaltsatou et al., 2011	Yes	Yes	No	No	No	No	No	No	Yes	Yes	Yes	4
Rogers et al., 2016	Yes	Yes	Yes	Yes	No	No	Yes	No	Yes	Yes	Yes	7
Soriano-Maldonado et al., 2022	Yes	Yes	Yes	Yes	No	No	Yes	Yes	Yes	Yes	Yes	8
Szalai et al., 2015	No	No	No	No	No	No	No	Yes	Yes	Yes	Yes	4

Items: (1) eligibility criteria were specified; (2) subjects were randomly allocated to groups; (3) allocation was concealed; (4) the groups were similar at baseline; (5) there was blinding of all subjects; (6) there was blinding of all therapists; (7) there was blinding of all assessors; (8) measures of at least one key outcome were obtained from more than 85% of the subjects who were initially allocated to groups; (9) intention- to- treat analysis was performed on all subjects who received the treatment; (10) the results of between-group statistical comparisons are reported for at least one key outcome; (11) the study provides both point measures and measures of variability for at least one key outcome; total score: each satisfied item (except the first) contributes 1 point to the total score, yielding a PEDro scale score that can range from 0 to 10

Table 4 shows the scores in the Evidence Project risk of bias tool. The maximum score of the scale is 8, with a mean scale score of 6 and a mean SD of 0.89. Two studies scored only 5, whereas another two studies achieved a score of 7. All the interventions chosen fulfilled the items of having a control group, a follow-up and similar socio-demographic characteristics in both groups at the start of the intervention. Five of the six studies met the items of randomisation of the groups, and five of the six articles presented pre-intervention values.

**Table 4:** Evidence project risk of bias tool

Estudio/item	1	2	3	4	5	6	7	8	Score
Campbell et al., 2005	Yes	Yes	Yes	Yes	No	Yes	Yes	Yes	7
Courneya et al., 2003	Yes	Yes	Yes	Yes	No	Yes	Yes	No	6
Kaltsatou et al., 2011	Yes	Yes	No	Yes	Yes	Nr	Yes	Nr	5
Rogers et al., 2016	Yes	Yes	Yes	Yes	No	Nr	Yes	Yes	6
Soriano-Maldonado et al., 2022	Yes	Yes	Yes	Yes	No	Yes	Yes	Yes	7
Szalai et al., 2015	Yes	Yes	Yes	No	No	Yes	Yes	No	5

Items: (1) Cohort; (2) control or comparison group; (3) pre/post-intervention data; (4) random assignment of participants to the intervention; (5) random selection of participants for assessment; (6) follow-up rate of 80% or more; (7) comparison groups equivalent on sociodemographics; (8) comparison groups equivalent at baseline on outcome measures

### Participants characteristics

The baseline characteristics of the participants are shown in Table 5. The total sample size was 535 individuals. The exercise and control groups were formed by 269 and 266 participants, respectively. In five articles, the sample consisted exclusively of females [50–54], whereas Courneya et al. [55] indicated that the sample consisted of 84.4% females. The mean age of the participants was 52.05 (47–57) years, with a mean (SD) of 51.94 (3.21) years in the exercise group and 52.16 (3.33) years in the control group. Regarding the type of cancer, some articles included only persons with breast cancer or individuals who have overcome breast cancer [50–53]. Courneya et al. [55] reported that participants had overcome different types of cancer such as breast, colon, ovarian, stomach and melanoma, Hodkin’s disease, non-Hodkin’s lymphoma, brain and lung cancer. Szalai et al. [54] indicated that some participants (65.9%) were mostly experiencing breast cancer, and the remaining ones (34.1%) were patients with other types of cancer. Two interventions took place during treatment [50, 55], while three interventions were performed after treatment [51–53]. Two articles reported the participants’ body mass index (BMI), with a mean of 28.75 (1.94), being 29.10 (2.40) in the exercise group and 28.40 (2.97) in the control group. Finally, regarding the level of physical activity, all studies included participants who were generally not highly trained.

**Table 5:** Baseline characteristics of participants

**Study**		**Design**	**Group**	**Sample size**		**Age mean (SD)**	**Gender**	**Cancer type (%)**	**Treatment (%)**	**Timing**	**Stage**	**BMI**	**Physical activity level**	
**Campbell et al., (2005)**		RCT pilot	EG	10		48 (10)	Female	Breast cancer	Radiotherapy or chemotherapy	During	NR	NR	< 3 times per week during 20 min of vigorous exercise	
			CG	9		47 (5)								
**Courneya et al., (2003)**		RCT	EG	51		51.55 (10.15)	84,4% females	Breast (40%), colon (9.4%), ovarian (5.2%), stomach and melanoma (4.2%), Hodkin´s disease, non-Hodkin´s lympoma, brain and lung cancer (3.1%), other cancers (15.6), missing (8.3%)	Surgery (79.5%), radiation (50%), chemotherapy (68.2%)	During (34.1% chemotherapy, 19.3% radiation and 44.3% at least one treatment)	50 % stage I/II; 50% stage III/IV	NR	Weekly minutes of PA: 192.53 (227.43)	
			CG	45									Weekly minutes of PA: 137.68 (117.76)	
**Kaltsatou et al., (2011)**		RCT	EG	14		56.6 (4.2)	Female	Breast cancer	Surgery, radiotherapy and chemotherapy	After (at least 3 months)	NR	NR	Sedentary	
			CG	13		57.1 (4.1)								
**Rogers et al., (2016)**		RCT	EG	110		54 (9)	Female	Breast cancer	Surgery, chemotherapy (58%), radiation (68%), hormonal therapy (49%)	After (surgery, chemotherapy and radiation) and during (hormonal therapy)	DCIS (11%), stage I (42%), stage II (35%), stage III (12%)	30.8 (6.9)	<30 min of vigorous or <60 min of moderate intensity physical activity per week	
			CG	112		54 (9)						30.5 (6.8)		
**Soriano-Maldonado et al., (2022)**		RCT	EG	29		52.6 (0.16)	Female	Breast cancer	Surgery, radiotherapy or chemotherapy	After	NR	27.4 (4.2)	<300 min per week	
			CG	28		52.0 (0.19)						26.3 (5.3)		
**Szalai et al., (2015)**	CT		EG	55	48.87 (1.2) SE		Female	Breast (69.1%) and other cancer (30.9%)	NR	Patients in different treatment periods	Primary (67.3%); Metastasic (32.7%)	NR		NR
			CG	59	51.31 (1.4) SE			Breast (62.7%) and other cancer (37.3%)			Primary (64.4%); Metastasic (35.6%)			

*RCT* randomised control trial, *RCT pilot *Pilot randomised control trial, *CT *control trial, *EG *exercise group, *CG *control group, *SD *standard deviation, *NR *non-reported, *DCIS *ductal carcinoma in situ

### Intervention characteristics

Table 6 describes the exercise protocols included in the meta-analysis. The mean duration of the interventions was 19.67 (14.77) weeks. Three of them lasted 12 weeks [50, 52, 53]. Other interventions’ durations were 10 weeks [55], 24 weeks [51] and 48 weeks [54]. Interventions consisted of exercising for one [54], two [50, 53] and three or more sessions per week [51, 52, 55]. Thus, the mean frequency of weekly sessions was 2.50 (1.05) sessions. The mean duration of the exercise sessions was 53.50 (25.84) min. Sessions’ durations ranged from 15 to 50 min in shorter sessions [52, 55], and from 60 to 90 min in longer ones [51, 53, 54]. In the exercise group, aerobic, resistance and combined aerobic and resistance exercise were performed in three [52, 54, 55], one [53] and two [50, 51] studies, respectively. The mean intensity of aerobic exercise was 65.50 (40–80) % of maximum heart rate [HRmax]. There were lower-intensity interventions (40–59% HRmax) [52], as well as higher-intensity interventions (65–80% HRmax) [50, 51, 55]. Exercise intensity ranged from 40 to 70% of one-repetition maximum (1RM) during the resistance exercise intervention that reported it [53]. Concerning the control group, most studies continued with their usual care, without any exercise or physical activity intervention [50–52, 54]. In other interventions, the control group performed ≥10,000 steps [53] and psychotherapy sessions [55]. In these cases, the exercise group performed the same activities as the control group to isolate the effect of the exercise intervention.

**Table 6:** Duration, frequency, attendance and intensity of exercise interventions

Study	**Group**	**Duration**	**Sessions duration/ frequency**	**Exercise description**	**Intensity progression and control**	**Adherence**
Campbell et al., (2005)	EG	12 Weeks	2 times per week	Warm-up	65-75% HRmax	Attendance: 70% (20)
				Exercise: 10-20 min walking, cycling, low-level aerobics, muscle-strengthening exercises.		86.36% completed intervention
				Cool down and relaxation		
	CG	12 Weeks		Usual care		
Courneya et al., (2003)	EG	10 weeks	Phychotherapy classes: 90 min / once per week	Psychotherapy classes + aerobic exercise (walking, swimming or cycling)	65-75 % HRmax	Completed intervention: 85%
			Exercise: 20-30 min / 3-5 times per week			
	CG	10 weeks	Phychotherapy classes: 90 min / once per week	Psychotherapy classes		Completed intervention: 94%
Kaltsatou et al., (2011)	EG	24 weeks	60 min/ 3 times per week	Warm-up: range of motion exercises and stretching	65-80% HRmax	NR
				Aerobic training: Greek traditional dances (each dance 3-4 min and with 15s breaks).		
				Resistance training: upper body execise		
				Cool down: stretching		
	CG	24 weeks		Usual daily schedule		NR
Rogers et al., (2016)	EG	12 weeks	15-50 min / ≥3 times per week	From 45 to +150 min/week of aerobic exercise (i.e. walking) at 40-59% HRmax	Duration: 45-75 to ≥150 min per week	NR
					Intensity: 1.5-3 to 3.5-5.5 RPE (40-59%HRmax)	
	CG	12 weeks		Usual care		
Soriano-Maldonado et al., (2022)	EG	12 weeks	60 min / 2 times per week	≥ 10,000 steps/d	Warm-up: aerobic exercise (50-65%HRR); 3/10 OMNI scale	Attendance: ≥75%
				Phase 1 (2 weeks): familiarization (individual needs and limitations)	Resistance training: 40-70% 1 RM	
				Phase 2 (10 weeks): Warm up (aerobic exercise, mobility) + resistance training (4 dynamic exercises) + cool down (stretching)	Cool down: dynamic/static stretching	
	CG	12 weeks		≥ 10,000 steps/d		
Szalai et al., (2015)	EG	48 weeks	Belly dance: 90 min / 1 time per week	Instructed belly dance	NR	NR
			Free interaction session: 90 min / 1 time per week	Free interaction session: discuss current problems (body image, sexuality, social relationship…)		
	CG	48 weeks				

*EG *exercise group, *CG *control group, *min *minutes, *s *seconds, *d *day, *HRmax *maximum heart rate, *HRR *heart rate recovery, *1RM *one-repetition maximum, *NR *non-reported

### Satisfaction with life measures

Life satisfaction was measured in the included studies with different scales. In most studies [50, 52, 53, 55], the “Satisfaction with Life Scale” (SWLS) was used. This questionnaire has five items with questions related to how similar their real life is compared to their ideal life, assessing their satisfaction with life [22]. Each question has a score ranging from 1 to 7, so the overall score of the questionnaire ranges from 5 to 35, the higher the score, the higher the satisfaction. This scale is a valid and reliable tool to assess this domain, which is related to HRQoL [27]. Kaltsatou et al. [51] used the “Life Satisfaction Inventory” (LSI), a 13-item scale that assesses participants’ satisfaction with their lifestyle [45]. Szalai et al. [54] used subscale D1 of the Campbell questionnaire, with scores ranging from 1 to 10, assessing satisfaction or frustration with life during the patient's illness.

### Overall results

**Fig. 2:**
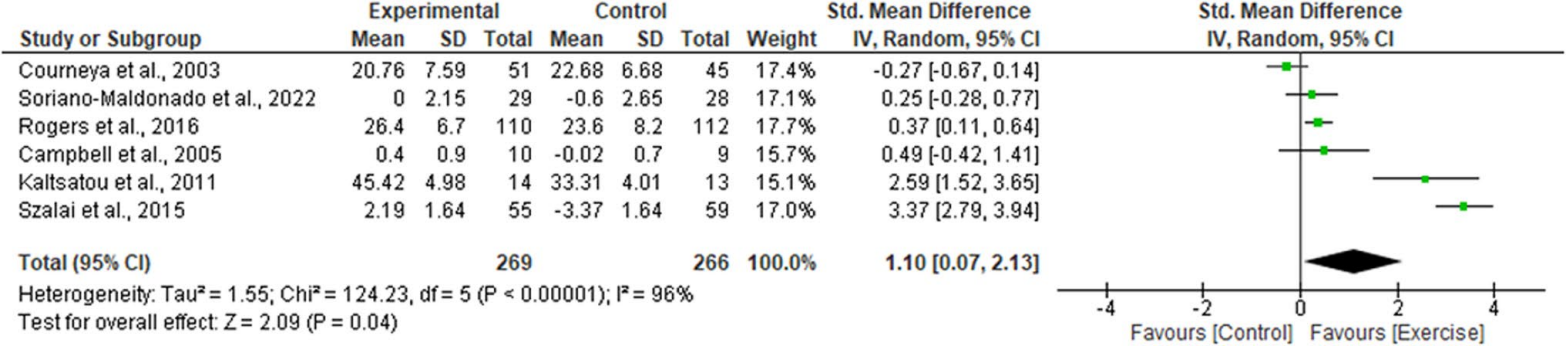
Effects of exercise on life satisfaction as compared to control. The forest plot shows standardized mean differences with 95% confidence intervals (CI). The diamond at the bottom of the graph represents the pooled standardized mean difference with 95% CI for all trials following random effects meta-analyses. The size of the plotted squares reflects the relative statistical weight for each study

The overall results of life satisfaction are shown in Fig. 2. The present meta-analysis shows that the individuals enrolled in the exercise programs reported an enhancement in the score ratings of life satisfaction rates higher than individuals included in non-exercise control groups (*p* = 0.04), with an SMD of 1.10; with a 95% CI of 0.07 to 2.13. The SMD shows a large effect size (> 0.75).

## Discussion

The aim of the present systematic review and meta-analysis was to analyse the effects of physical exercise programs on life satisfaction in patients and survivors of cancer. The main finding was that physical exercise interventions of between 10 and 48 weeks, with a session length of between 15 and 90 min, an intensity of between 40 and 80% HRmax in aerobic exercise and an intensity of between 40 and 70%1RM in resistance exercise, could improve overall life satisfaction in individuals with cancer and persons who have overcome cancer. This outcome was obtained from the meta-analysis of six controlled trials that compared an exercise intervention in patients and survivors of different typologies of cancer with a group of patients with similar characteristics that received only the usual care for cancer. Out of the six controlled trials included in the meta-analysis, five showed higher mean values of life satisfaction after the exercise program or higher improvements in life satisfaction ratings between pre- and post-exercise program measurements with respect to the non-exercise intervention control group. However, only three of them demonstrated statistically significant differences. Additionally, only one of the trials [55] showed higher mean values of post-intervention life satisfaction in the control group than in the exercise group, although without significant differences. The result of this latter trial [55] can be explained by the circumstance that, despite being a randomised study, there were significantly higher pre-intervention life satisfaction values in the control group than in the exercise group. Overall, despite a certain variability in the results of the studies included in this meta-analysis, the positive results for life satisfaction found in the patients with cancer enrolled in the exercise programs and the large effect size might suggest that a program of physical exercise could be an effective tool to improve life satisfaction in individuals with cancer and in persons who have overcome cancer. Hence, health and physical exercise professionals working with these persons may consider setting up a program of physical exercise to enhance low-life satisfaction. This may be a convenient strategy to improve the overall status of people diagnosed with cancer as exercise has been deemed as effective to enhance HRQoL [30], in addition to the potential improvements in life satisfaction reported here.

The quality of the trials, which included an appropriate sample size for each group and standardizations to allow a comparison between groups that only differed in the exercise intervention, reinforced the results about the potential effect of regular exercise on improving life satisfaction in persons diagnosed with cancer. This outcome is in agreement with other meta-analyses indicating that exercise programs could improve life satisfaction in healthy people [56]. In terms of the possible relationship between life satisfaction ratings and HRQoL, the present results are in line with those of other meta-analyses indicating that physical exercise programs significantly could improve HRQoL in individuals with cancer and persons who have overcome cancer [29–32]. Thus, setting a program of exercise might improve life satisfaction in people diagnosed with cancer, and it may be a co-adjutant of other therapies to improve the prognosis of the illness and reduce mortality [28]. Several mechanisms could explain the potential positive effect of an exercise program on life satisfaction in persons diagnosed with cancer as regular exercise contributes to the reduction of stress [37], symptoms of depression and anxiety [37], sleep disorders [37], cancer-related fatigue [38] and pain [39]; and improves self-esteem [40], body image [37] and social functioning [37]. All of these benefits of exercise in individuals diagnosed with cancer may contribute to an increase in life satisfaction although further investigation is needed to determine which of these factors contributes more to an overall enhancement of life satisfaction in these individuals. This potential improvement in perceived life satisfaction with exercise may be particularly relevant because of the severe negative effects caused by the disease and its treatments, which could lead to reduced life satisfaction after diagnosis [20]. Improving life satisfaction could be important in these patients because of its relationship with lower levels of symptoms of depression and anxiety [57], and with a decreased risk of suicide [25, 26]. In addition, improved life satisfaction could be associated with better acceptance of the disease [58] and better survival prognosis in patients with cancer [28].

The current meta-analysis included aerobic, resistance and combined aerobic and resistance exercise interventions. Both exercise modalities may lead to certain benefits in persons diagnosed with cancer that could be related to improvements in life satisfaction. On the one hand, resistance exercise may increase muscle mass [59], strength [59] and muscle power [60], which may be especially important among patients undergoing cancer treatments, such as chemotherapy, as these treatments may negatively affect muscle strength and muscle power [61]. Furthermore, these positive effects of resistance exercise may be associated with a decrease in cancer-related fatigue [60], which could indirectly contribute to an increase in patient’s life satisfaction [62]. On the other hand, aerobic exercise has benefits including the reduction of common symptoms such as pain, insomnia, fatigue and dyspnoea [63]. In addition, dyspnoea caused by treatments could lead to symptoms of stress and depression [64]. Therefore, the reduction of all these symptoms through aerobic exercise might lead to an improvement in patients’ life satisfaction. Thus, both aerobic and resistance exercise could contribute to the improvement of life satisfaction in individuals diagnosed with cancer.

Due to the small number of articles included in this meta-analysis, it was not possible to create subgroups according to exercise modality. Thus, further research is needed to find out which type of exercise is the most effective in order to improve patients’ life satisfaction. In this sense, the interventions included in the present meta-analysis were very heterogeneous and included running, walking, cycling, swimming, resistance exercise or dancing. Among all these types, the two interventions with the largest effect sizes involved dance interventions (belly dance and Greek traditional dance). In other populations, such as older adults, it has also been shown that dance could be an interesting strategy to improve life satisfaction [65]. Therefore, aerobic dance-based exercise may be an interesting strategy to improve life satisfaction in cancer individuals with cancer and survivors. However, this statement should be taken with caution, as only two of the studies included in the present meta-analysis involved dance. Thus, more research is needed to study the effect of dance on life satisfaction in persons diagnosed with cancer. Another consideration is that a substantial proportion of the studies included in the review focused on interventions administered to patients diagnosed with breast cancer. Consequently, further research with patients affected by other types of cancer is warranted to improve understanding of the potential effectiveness of exercise interventions to improve life satisfaction, specifically tailored to different types of cancer.

The main limitation of the present meta-analysis is that the number of trials studying whether physical exercise programs improve life satisfaction in persons with cancer and individuals who have overcome cancer is low. Only six articles were included in the present systematic review and meta-analysis. In addition, one of the included studies was not randomised [54], and another one presented relevant baseline differences in life satisfaction [55], which reduced the overall effect size reported in the meta-analysis. Although five of the six included articles showed higher values of life satisfaction after the exercise programme, only three of them showed statistically significant differences. Hence, a notable limitation of the current meta-analysis is the variability of the results between the included studies. Furthermore, it is crucial to highlight the considerable heterogeneity observed among the studies included in the meta-analysis, which may explain the variability among the results. This heterogeneity is manifested in several aspects, including the diversity of the studied populations, as both patients with cancer and survivors of cancer have been included. Moreover, the type of cancer of the participants is not consistent, as interventions based on different types of cancer have been included. In addition, the types of exercise in the programmes are not heterogeneous, with different types of exercise modalities. Based on the results of the current study, a greater number of interventions are needed to identify the most suitable exercise type to improve life satisfaction. In addition, there is a need for higher quality research (randomised controlled trials) with similar baseline values for the life satisfaction variable and with control and experimental groups only differing in whether they take part in the exercise intervention. Another limitation is that most participants in the included studies were women, so the findings of the present review may be more applicable to females, without knowing whether they may be extrapolated to male individuals. In this regard, if the meta-analysis was conducted exclusively with the five articles comprising only female participants, the size of the effect of exercise on life satisfaction would be even amplified, increasing the SMD of the exercise-control comparison from of 1.10 to 1.39. Further investigation is warranted to ensure that the effect of exercise on life satisfaction in individuals with cancer is comparable in men and women. Last, another limitation of the current meta-analysis is that none of the included articles focused on a particular ethnic group. In this sense, it would be interesting to conduct future scientific research focused on analysing the impact of physical exercise on individuals diagnosed with cancer from diverse ethnic backgrounds.

## Conclusions

Physical exercise programmes might potentially enhance life satisfaction in individuals with cancer and persons who have overcome cancer. This could be associated with a better prognosis and self-esteem and with less stress, anxiety, depression and frustration. These findings may provide insight for health and physical exercise professionals to use physical exercise as a potential tool to combat the low life satisfaction often experienced by persons diagnosed with cancer. Given the low number of studies and the heterogeneity in methods and participants, interpretation of results must be done with caution and more quality research is needed in this area.

## Data Availability

The data that support the findings of this review are available on request from the corresponding author.
